# Adapting the Gamified Educational Networking (GEN) Learning Management System to Deliver a Virtual Simulation Training Module to Determine the Enhancement of Learning and Performance Outcomes

**DOI:** 10.7759/cureus.26332

**Published:** 2022-06-25

**Authors:** Samira Wahab, Adam Dubrowski

**Affiliations:** 1 Health Sciences, Ontario Tech University, Oshawa, CAN; 2 Faculty of Health Sciences, Ontario Tech University, Oshawa, CAN

**Keywords:** simulation-based education, simulation assessment, student education, microtomy, simulation training

## Abstract

Microtomy is a medical laboratory sciences procedure that medical laboratory technologists (MLTs) use to cut tissues for microscopic examination. Due to safety concerns and the potential to destroy tissue samples, learners must perform the procedure correctly. In order to allow for safe and controlled learning, this procedure should be conducted in a simulated setting before attempting with human tissues.

The objective of this study is to describe the development and user-based evaluation of a virtual simulation training module. A research group developed the virtual simulation training module's content and design, and a local MLT expert provided the content. Nine students enrolled in a university-based medical laboratory sciences program provided feedback about the module. The results demonstrated that the virtual simulation training module was an effective and user-friendly learning tool for the medical laboratory sciences program. Although more validity and efficacy testing are required in the future, the students indicated a potential to use this module to prepare future students for hands-on exercise in a simulation laboratory setting.

## Introduction

Medical laboratory technologists (MLTs) perform the microtomy procedure to cut tissue for further examination, making this a critical process for any sample slide examination under the microscope [1]. When conducting the microtomy procedure, it is vital to understand the procedure's steps and safety features. If a tissue sample is prepared incorrectly, the entire tissue sample is tarnished and must be prepared again [2]. A series of steps must be completed in sequential order, and it is crucial that learners must become aware of the cautious safety features when handling the instrument [2].

Simulation-based education is an educational practice that introduces learners to an extensive learning environment allowing them to develop skills and receive feedback without the pressure and cost of errors [3]. When practicing in a simulated setting, the trainees can develop the skills required at their own pace with unlimited repetition of specific scenarios catered to the skill [3].

Despite simulation-based learning being introduced into medical laboratory sciences, its systematic use is underutilized [3]. Simulation methods are ideal for optimizing the knowledge and skills of trainees with guided experiences before they are trusted with real patients [3]. However, simulation is expensive [4] and logistically difficult to organize [4]. Literature suggests that pre-simulation preparedness leads to more effective simulation [4]. Although didactic and/or reading assignment-based preparations are predominant, early evidence from our laboratory suggests that more experiential learning opportunities may be more effective in accomplishing this goal [5]. Therefore, this technical report aimed to describe the design and preliminary evaluation of a pre-simulation, virtual simulation module that students can complete before coming to the simulation laboratory for hands-on learning.

## Technical report

Medical laboratory sciences students complete a four-year university-based program to obtain a bachelor’s degree. During their program, they must undergo histology courses in their third year to learn the microtomy procedure. In the medical laboratory sciences program, the students learn the microtomy procedure in both lectures and the laboratory in the first semester of their third year. The students are also offered one revision lesson in practicing their microtomy skills in the second semester of their third year. The virtual simulation module was designed as an educational tool for medical laboratory students to perform the microtomy procedure. The intended learners are students of the medical laboratory sciences program who have undergone traditional laboratory learning.

Inputs and design process

The virtual simulation module was initially discussed by a research team that focuses on promoting knowledge using simulation as the approach and a medical laboratory expert regarding the format and layout of the module’s presentation. This happened using a series of interviews and iterative redesign cycles. The expert was an MLT who obtained their Medical Laboratory Technology diploma from the Michener Institute of Applied Health Sciences in Toronto, Ontario. The expert has also completed their Clinical Research Associate certificate at Michener Institute, received a Master of Health Studies from Athabasca University, Athabasca, Canada, and is currently an associate teaching professor at Ontario Tech University, Oshawa, Canada. Communication between the researcher and the expert was conducted via email and Google Meets. Email communication was used to set up meetings, and Google Meet is where the feedback was discussed regarding the layout and appearance of the module. Once the changes were made, an email was sent to confirm if the changes were appropriate and if further edits needed to be made. However, after the first edits, the virtual simulation training module was deemed appropriate by the expert. The total time of the study took a few months from developing the module and completing the testing.

The virtual simulation module is held on a gamified educational network (GEN), an online learning management system that supports and administers training programs and educational courses. The platform provides gamification elements such as a leaderboard system where participants can rate the quality of others’ comments and interactions [6]. The leaderboard system shows the ranking in a private version that does not show the rest of the users who are at each position, which avoids comparisons that could be detrimental to motivation. GEN also has a feedback feature for the learner to receive constructive feedback, an essential part of an online learning experience. The feedback can consist of peer-to-peer and expert-based feedback [6]. This type of computer-based video training (CBVT) is an emerging method of self-directed learning method that allows learners to take the initiative and responsibility for their learning [7]. It is a portable, convenient, flexible, and consistent form of learning that is inexpensive once implemented [7].

Communication with a computer science expert from Ontario Tech University, who was also a designer of the virtual simulation module, was conducted via email. The requirements of the content and design based on the MLT expert’s feedback were written out, and the computer science expert completed the task. A link was then created via email to view the module and all of its features, and when providing feedback, it was written out again via email. Once the changes were made, after consulting the MLT expert, another link was provided by the computer science expert to view the module, and the second time, it was deemed adequate. Essentially, the expert was the input of the ideas of the content and visuals of the module, and the computer science expert provided the outputs.

Products/outcomes

To assess the usability of the system after the module was completed, all participants provided their feedback using the system usability scale (SUS) questionnaire, which allowed us to (a) assess their perceptions in relation to the module’s content, (b) assess the module’s potential to serve as an educational tool, and (c) provide possible improvements to the module. SUS has been shown to effectively distinguish between unusable and usable systems or better than proprietary questionnaires [8]. The purpose of using SUS is that it is an easy scale to administer to small sample sizes with reliable results [8]. The participants were asked to score a list of 10 items (Table 1) using a (1-5) Likert scale and provide open-ended feedback about the module and suggestions for possible improvements.

**Table 1:** System usability scale (SUS) questionnaire

Educational Value
1. I think that I would like to use this frequently.
2. I found this unnecessarily complex.
3. I thought this was easy to use.
4. I think that I would need assistance to be able to use this.
5. I found the various functions in this were well integrated.
6. I thought there was too much inconsistency in this.
7. I would imagine that most people would learn to use this very quickly.
8. I found this very cumbersome/awkward to use.
9. I felt very confident using this.
10. I needed to learn a lot of things before I could get going with this.

Virtual simulation module

The virtual simulation module begins by providing the background and objectives of the module followed by key microtome safety instructions. Once the participants had read through these sections, they moved on to the four learning objectives. Each learning objective begins with a description of what that section is about and is followed by a video that they must watch and a question that they must answer correctly. Once the participants answer the question, the correct answer is shown, along with a description of why the correct answer is correct. If the student chooses the incorrect answer, the correct answer is shown along with an explanation as to why the correct answer is correct. Formative feedback is provided during the course of an assessment, such as the learner completing the quiz portion of the virtual simulation module. Constructive feedback is provided after each section of the module; thus, the learner is not overwhelmed with the amount of feedback. The participant cannot move through the sections until the previous section has been completed.

**Figure 1:**
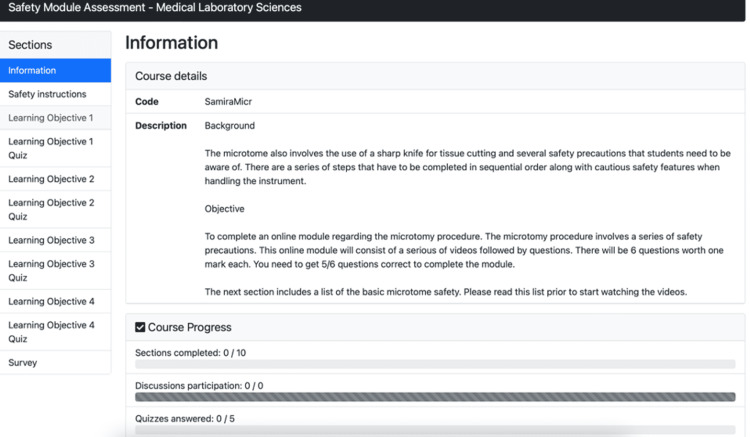
Virtual simulation training module introduction and landing page

Figure 1 illustrates the GEN virtual simulation module and shows the introduction and landing page, which is the first page that appears after the participant has logged in.

**Figure 2:**
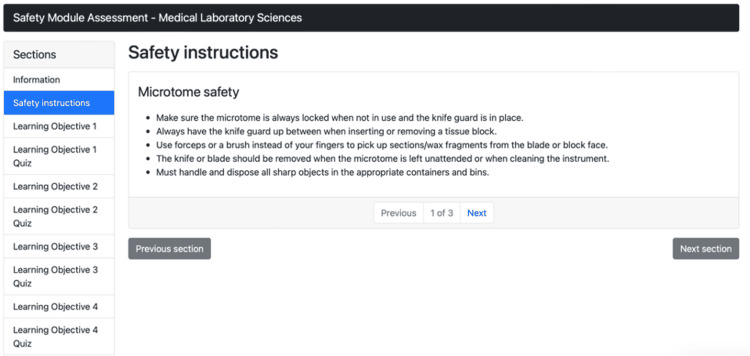
Virtual simulation training module safety instructions

Figure 2 illustrates that the GEN virtual simulation module shows the first of three pages of the safety instructions for the microtomy procedure. The first page lists key microtomy safety instructions, the second page shows a labeled diagram of the microtome, and the last page shows the conclusion and the marked completed section.

**Figure 3:**
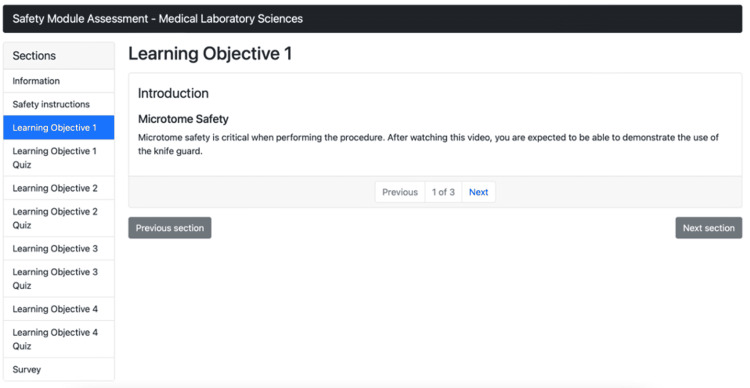
Virtual simulation training module learning objectives

Figure 3 illustrates the GEN virtual simulation module and shows an example of the learning objectives for the module. There are a total of four learning objectives for this module.

**Figure 4:**
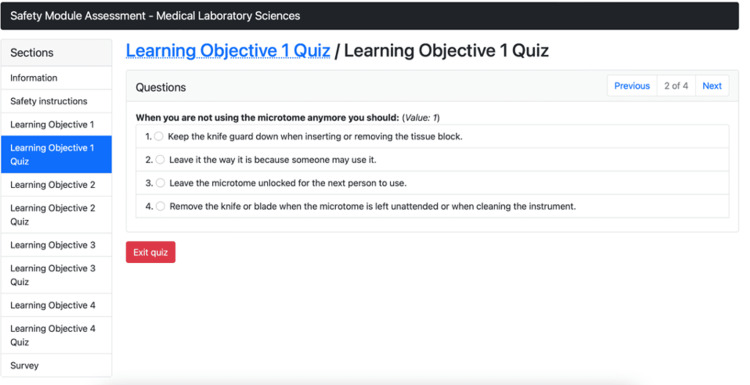
Virtual simulation training module quiz example

Figure 4 illustrates the GEN virtual simulation module and shows the example of the quiz question that appears after the participant has completed the material for the specific learning objective.

**Figure 5:**
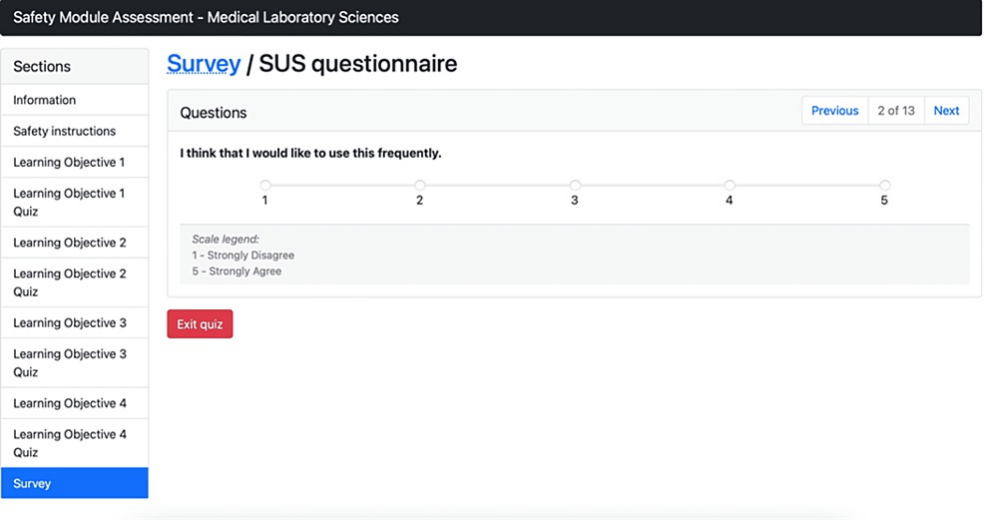
Virtual simulation training module SUS questionnaire SUS: System usability scale.

Figure 5 illustrates the GEN virtual simulation module and shows an example of the SUS questionnaire, where the participants can rate the module.

Assessment of the simulator

Nine medical laboratory students from Ontario Tech University were asked to complete the module virtual simulation training module delivered through the GEN platform [9]. GEN consists of various features to increase motivation by incorporating game elements and peer-to-peer collaboration [9]. However, for the purpose of this technical report, these features were disabled, and the GEN platform provided the video and quiz features to the participants.

User assessments

All nine participants completed the survey. Overall, the majority of the participants rated the module to be above average (Table 2). Based on previous SUS score studies, a SUS score of 68 and above would be considered above average, and anything below 68 is below average [8]. Scores above 80.3 are considered to be in the top 10% of scores [8]. Therefore, the scores that were obtained demonstrate that all nine participants rated the module as above average, and six out of nine participants have scored the module in the top 10% of scores.

**Table 2:** SUS scores from each of the participants SUS: System usability scale.

Participants	Scores
1	100
2	97.5
3	90
4	85
5	75
6	82.5
7	97.5
8	95
9	77.5

After the participants rated the module by completing the SUS evaluation, they also provided free-text feedback regarding the module. These comments are summarized in Table 3. In general, the virtual simulation module was perceived to be educational and easy to follow. Every participant commented that the module was easy to use. This is very important when the students complete the module as the students are completing the module remotely and in their own time. A couple of the comments suggested to revise some of the wording of the questions so that it reads more clearly.

**Table 3:** Free-text feedback from the participants after completing the module

Free-Text Feedback
Awesome. I like it.
These modules are very easy to follow and the quizzes per section helps ensure you understand before moving forward.
It was overall very helpful and helped solidify my knowledge on the microtome. I liked how the videos were broken up into using the microtome, safety, and how to clean the microtome.
There were some spelling mistakes. The wording was slightly confusing at times, but overall very easy to use and helpful for reviewing for our histo midterm next week.
It was easy to use, but the question answers were long to read.
After hitting the ‘mark completed’ button at the end of a learning objective section, it would send me back to the first slide of that learning objective. It would be more intuitive if that button sent me to the quiz for that section (linking it together so that each time I clicked “next” on the last slide of the previous section, it sent me to the first slide of the next section). The quiz questions were sometimes unclear exactly what was meant, e.g., The purpose of coarse trimming “to remove excess wax from around the tissue” would have been more clearly phrased as “to remove excess wax from around the surface of the tissue” (most of the wax around the tissue is supportive and is not removed). One of the microtomy questions contradicted the video, where the instructor stated the knife guard was not to be up while cutting the tissue or interact with the tissue block at all, but the question claimed the purpose of the knife guard was to protect the tissue while cutting as well as protect fingers. The clearly labeled diagram of a microtome was nicely done. In the video, it was difficult to see where the instructor was indicating from the positioning of the camera because her hand/arm was often blocking. The objectives broke down learning about the microtome into easy-to-understand pieces, and I think this would be a valuable tool to go through once before the first cutting lab. It would be valuable to add seeing how the microtome actually works (demonstrating how the blocks are cut) to put the pieces into context. This module seems most useful for students who have never seen a microtome before.
Very intuitive and easy to use. Feedback for corrections was a nice addition to the quizzes.
This module was designed really well. It was clear, quick, and easy to understand.
I found this platform very nice, and the layout is great for learning.

## Discussion

The rationale for developing the virtual simulation module presented in this technical report is to provide an educational tool for the microtomy procedure so that students can learn and practice the procedure remotely. Numerous studies have suggested that an online learning environment may possess the same learning benefits as face-to-face learning with an instructor present [4,6,10]. Having the learners use this module can increase their training opportunities and ultimately improve learning outcomes. Consequently, this report outlined how the virtual simulation module can be used as an educational tool for microtomy students.

After the participants used the virtual simulation module, the module received overall positive feedback. The participants considered it to be a useful tool to learn and practice microtomy. All of the participants reported that the module was above average demonstrating that it was clear and easy to follow. However, some participants found some minor issues. Specifically, some of the module questions could have been worded differently so that the question is clearer as a few of the participants found them to be confusing. The virtual simulation module presented microtomy in sections to learn the names of all the parts of the instrument, safety, cutting process, and cleaning. This allowed them to learn one section at a time until they had understood the material and answered the questions correctly before moving on to the next section. Despite some of the shortcomings of the module, all of the participants rated the module as above average and thought it was helpful for their learning process.

The participants collectively agreed that the module was clear and easy to follow concluding that the GEN platform was a useful platform with a clear layout to navigate. One of the student’s open-ended feedback items described that they liked the GEN platform and that the layout of the module is great for learning. Another participant also commented that they like how the module was broken down into sections, instead of having all the content provided at once.

The main limitation of the study was that there was no comparison group of learners who had no previous knowledge of microtomy to complete the module. The benefit of comparing the results of learners with no prior knowledge of microtomy is to determine how much learning was gained from the module without the traditional laboratory learning. Due to COVID-19, participant recruitment for this subgroup was either non-responsive or declined as the students were overwhelmed with the amount of current online learning they are required to do. Another limitation of the user-based feedback was that some of the wordings of the questions were confusing and could have been worded differently to be more clear of what the question was asking. Despite having these comments on how the module could have been improved, the module was still perceived as useful for student learning. The module's questions and answers are being examined to eliminate grammar errors and any unclear wording of sentences. This change will improve and provide a clearer understanding to the participant of what the question is asking.

Based on the feedback provided by the students, the virtual simulation module would need some minor modifications before it can be considered for use in training. Although it does reach its objective, which is to allow the learner to practice the microtomy procedure before having to perform it in a clinical setting, the suggested improvements of the module will improve the understanding of the questions.

## Conclusions

The virtual simulation module that was designed as an educational tool was demonstrated to be an effective and useful tool for the microtomy procedure in addition to being the first virtual simulation module designed specifically for microtomy to our knowledge. We believe our module, with a few minor improvements, could become a valuable educational tool for students to practice the microtomy procedure in a remote environment.
